# Towards Understanding the Role of the Glycosylation of Proteins Present in Extracellular Vesicles in Urinary Tract Diseases: Contributions to Cancer and Beyond

**DOI:** 10.3390/molecules29225241

**Published:** 2024-11-06

**Authors:** Magdalena Wilczak, Magdalena Surman, Małgorzata Przybyło

**Affiliations:** 1 Department of Glycoconjugate Biochemistry, Institute of Zoology and Biomedical Research, Faculty of Biology, Jagiellonian University, Gronostajowa 9 Street, 30-387 Krakow, Poland; magdalena.wilczak@doctoral.uj.edu.pl (M.W.); magdalena.surman@uj.edu.pl (M.S.); 2 Doctoral School of Exact and Natural Sciences, Jagiellonian University, Prof. S. Lojasiewicza 11 Street, 30-348 Krakow, Poland

**Keywords:** bladder cancer, extracellular vesicles, glycosylation, prostate cancer, renal cancer, urinary tract, urine

## Abstract

Extracellular vesicles (EVs) are a population of nanoscale particles surrounded by a phospholipid bilayer, enabling intercellular transfer of bioactive molecules. Once released from the parental cell, EVs can be found in most biological fluids in the human body and can be isolated from them. For this reason, EVs have significant diagnostic potential and can serve as an excellent source of circulating disease biomarkers. Protein glycosylation plays a key role in many biological processes, and aberrant glycosylation is a hallmark of various diseases. EVs have been shown to carry multiple glycoproteins, but little is known about the specific biological roles of these glycoproteins in the context of EVs. Moreover, specific changes in EV glycosylation have been described for several diseases, including cancers and metabolic, cardiovascular, neurological or kidney diseases. Urine is the richest source of EVs, providing almost unlimited (in terms of volume) opportunities for non-invasive EV isolation. Recent studies have also revealed a pathological link between urinary EV glycosylation and urological cancers, as well as other pathologies of the urinary tract. In this review, we discuss recent research advances in this field and the diagnostic/prognostic potential of urinary EV glycosylation. In addition, we summarize common methods for isolating EVs from urine and techniques used to study their glycosylation.

## 1. Introduction

Research on biomarkers present in physiological fluids focuses on finding new biomarkers, increasing the sensitivity of their detection, developing more efficient sample preparation techniques, and introducing new, easier and less expensive analytical methods. This research area focuses mainly on samples derived from blood and serum, but cerebrospinal fluid and urine are also good media for diagnosing multiple diseases. Urine is produced in the kidneys and concentrates/accumulates metabolic products, especially nitrogen-containing substances that must be constantly removed from blood. Water, salts, urea, organic compounds, proteins, hormones and a wide range of other metabolites constitute about 95% of urine. The composition of urine largely reflects the proper functioning of the kidneys and other organs of the urinary tract. All of these characteristics make urine an excellent diagnostic material [1,2,3,4,5], along with the fact that obtaining a urine sample is risk-free, and the disposable sample volume available allows for multiple analyses to be performed simultaneously from a single sample.

Recently, extracellular vesicles (EVs) have been studied in terms of many aspects, including their role in physiological processes (e.g., their involvement in immune response [6] and coagulation [7]) as well as pathological processes (e.g., their role in cancer [8] and in autoimmune [9], neurodegenerative [10] and viral diseases [11]). At the same time, EVs are being explored as potential diagnostic tools [12,13] and therapeutic agents [14,15], including as drug delivery systems [16]. EVs are lipid-bilayer-enclosed nanostructures released by all cell types [17]. Three main populations of EVs are commonly distinguished, i.e., exosomes, microvesicles (also called ectosomes) and apoptotic bodies. These three subpopulations differ in biogenesis, size and molecular cargo. Exosome biogenesis begins with the invagination of the endosome membrane, resulting in the formation of multivesicular bodies (MVBs). Inside MVBs, small vesicles 30–100 nm in diameter are present. Fusion of MVBs with the cell membrane results in the subsequent release of exosomes into the intracellular space. Microvesicles are formed by the rearrangement of the cytoskeleton, resulting in the direct budding of the cell membrane and the release of vesicles 100–1000 nm in diameter. The release of apoptotic bodies is closely related to apoptotic cell death, during which the cell shrinks and produces vesicles with diameters of up to 5000 nm. A distinctive feature of apoptotic bodies is the presence of histones, DNA and even intact cell organelles in their cargo. Exosomal and microvesicular cargo both consist of various proteins (e.g., transcription factors, enzymes, transmembrane proteins, and receptors and their ligands), nucleic acids (e.g., mRNA, miRNA, lncRNA), metabolites and active forms of lipids [17]. Once released from cells, EVs are present in all physiological fluids and can be found in blood, cerebrospinal fluid, semen, breast milk, saliva and urine [18]. Finally, when using the presented classifications of EVs related to the biogenesis of particular populations, some of their limitations should be acknowledged. Common EV isolation methods do not separate EVs based on the mechanisms of their formation, but solely based on their size and/or density. The specificity of known exosomal and ectosomal markers is also being questioned. Therefore, the “Minimal information for studies of extracellular vesicles” (MISEV2023) guidelines suggest the operational use of the terms “small” and “large” EVs when characterizing isolated samples, with 200 nm being the discriminating diameter value. Similarly, for density-based isolation methods, the terms “low” and “high density EVs” are being used. Lastly, besides biogenesis-, size- and density-related nomenclature, more specialized EV classifications are also being introduced. Some of them relate to cellular processes or the cell type from which the EVs originate (e.g., cell migration—“migrasomes”; carcinogenesis (tumor cells)—“oncosomes”) [19].

**Figure 1:**
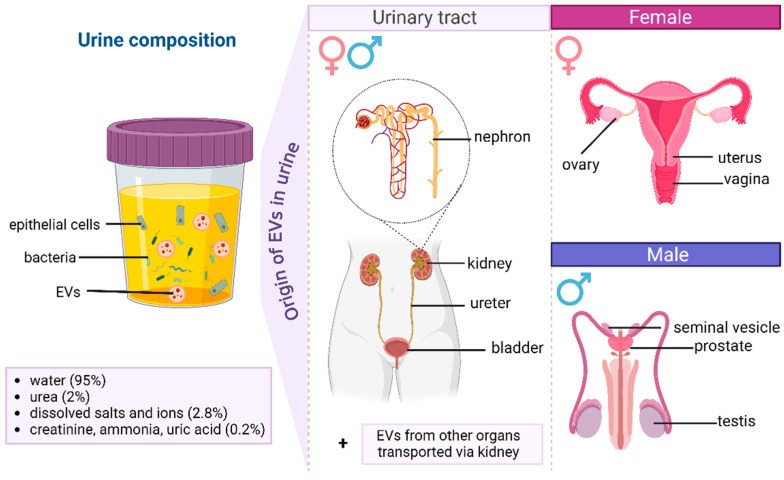
Main urine components and origin of urinary EVs.

Urine carries EVs derived from many organs of the urinary and reproductive systems (Figure 1). The majority of urinary EVs originate from the kidneys, ureters and bladder [20,21]. Thanks to well-known nephron biomarkers, it is possible to assign EVs to specific parts of the nephrons (e.g., Bowman’s capsule, the ascending or descending limb of the loop of Henle, and the proximal or distal convoluted tubule). Moreover, EVs present in women’s urine may also originate from the ovaries, uterus and vagina, while EVs originating from the prostate, testes and seminal vesicles can be found in men’s urine. However, urine can also transport EVs derived from other organs, as it also serves to purify blood, which contains EVs produced by other organs, even those distant from the urinary tract [20,21]. Biomarkers of prostate-, nephron- and bladder-derived EVs are well known (Table 1). However, biomarkers of EVs derived from the female reproductive system have yet to be determined.

As already mentioned, urine is the richest source of EVs, providing almost unlimited (in terms of volume) opportunities for non-invasive EV isolation. Recent studies have also demonstrated a pathological link between the glycosylation of urinary EV proteins and urological cancers, as well as other pathologies. In this review, we discuss recent research advances in this field and discuss the diagnostic/prognostic potential of urinary EV glycosylation. Additionally, we summarize the common methods used to isolate EVs from urine and the techniques used to study their glycosylation.

## 2. Glycosylation as One of the Post-Translational Protein Modifications Found in EVs

Glycosylation is one of the most common post-translational modifications of proteins, affecting their conformation, physicochemical properties and stability. Proper glycosylation determines the course of many physiological processes, such as cell adhesion, immune response, ligand–receptor interactions and many others. On the other hand, aberrant glycosylation is associated with the development of numerous diseases, including cancer [30]. Glycoconjugates are extensively distributed across outer and inner cell membranes, so EVs, as membrane-derived structures, are enriched in various glycan structures. Moreover, it has been shown that glycans are involved in the sorting of proteins into EVs, and the functional effect that EVs exert on recipient cells also depends on the glycosylation status of the parental cells [31,32].

### 2.1. Basic Information on Glycosylation

**Figure 2:**
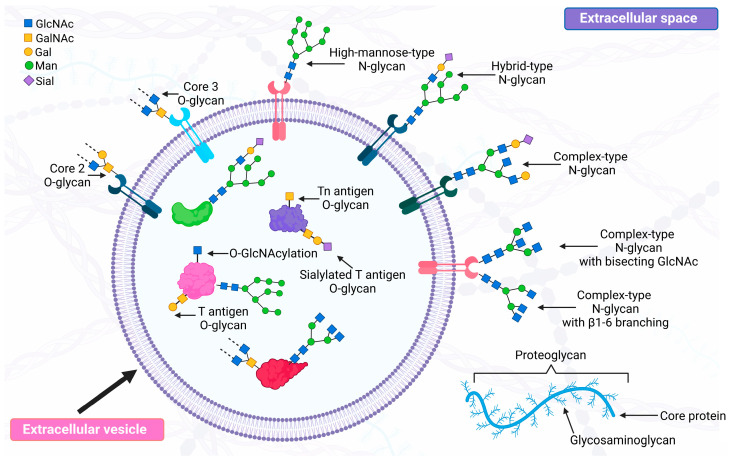
Major glycan structures found in EVs. GlcNAc—N-acetyloglucosamine; GalNAc—N-acetylogalactosamine; Gal—galactose; Man-mannose; Sial-sialic acid.

The two major types of glycosylation, i.e., *N*-glycosylation and *O*-glycosylation, differ in the final glycan structures formed (Figure 2). Asparagine residue (Asn) is crucial for *N*-glycosylation since *N*-glycans bind to the peptide through Asn present mostly in the Asn-X-Ser/Thr sequence, where X stands for any amino acid residue except that of proline. Though, sometimes this binding takes place through Asn in other amino acid sequences, for example, Asn-X-Cys. In addition, such an Asn residue should be on the surface of a protein, not in its interior. The synthesis of *N*-glycans is a multistep process and occurs successively in the cytoplasm, endoplasmic reticulum and Golgi apparatus. The process can be divided into three phases. In the first phase, a dolichol-linked oligosaccharide precursor (GlcNA_2_Man_9_Glc_3_) is synthesized by the addition of sugar residues in a specific order. In the second phase, the precursor is transferred *en bloc* to Asn in the appropriate sequences in the polypeptide chain. In the third phase, glycosidases remove some sugar residues, creating a pentasaccharide core (GlcNA_2_Man_3_) [33,34]. Further steps of *N*-glycosylation involve two groups of highly specific enzymes, i.e., glycosyltransferases and glycosidases, which are involved in the formation of high-mannose, complex and hybrid types of *N*-glycans by adding or removing appropriate sugar residues. In high-mannose *N*-glycans, only Man residues are attached to the core structure. Complex-type *N*-glycans are characterized by the presence of Man only in the core structure, which is expanded by the addition of other monosaccharide residues (GlcNAc, Gal, Fuc, Sial), which form so-called antennae. One of the most studied expansions of complex-type *N*-glycans is the addition of bisecting GlcNAc by β1,4 linkage to the core Man catalyzed by *N*-acetylglucosaminyltransferase III (GnT-III). The presence of bisecting GlcNAc inhibits the activity of *N*-acetylglucosaminyltransferase V (GnT-V), an enzyme, catalyzing the attachment of β1,6 GlcNAc to the core structure, forming β1,6-branched *N*-glycans that allow further formation of *N*-glycans with three or four antennae. Finally, hybrid-type *N*-glycans have features characteristic of both high mannose and complex-type *N*-glycans [33,34].

The synthesis of *O*-glycans also occurs in the endoplasmic reticulum, Golgi apparatus and sometimes in the cytoplasm, but is not as complex as the synthesis of *N*-glycans. *O*-glycans are linked by Thr/Ser residues to amino acid chains and do not possess one characteristic core structure [34]. High-molecular-weight mucins (MUCs) constitute the majority of *O*-linked glycoproteins. Most MUCs possess GalNAc at the protein’s reducing terminus. This GalNAc residue is known as Tn antigen, on which glycosyltransferases synthesize core 1, core 2 and core 3. Core 1, commonly named T antigen, is synthesized by the addition of Gal to Tn antigen. Core 2 is formed by adding GlcNAc to T antigen. Finally, core 3 is synthesized by adding two GlcNAc residues to Tn antigen. Further elongation of MUCs by successive linking of the sugar residues leads to the synthesis of eight more core structures. All of these core structures can be sialylated, fucosylated or sulfated to form structures such as sialyl Lewis^x^ or sulfo-sialyl Lewis^x^ [34,35].

Proteoglycans consist of a protein with one or more sugar side-chains, known as glycosaminoglycans (GAGs), attached to Ser or Thr residues in the polypeptide chain via a special linker. Repeating linear disaccharides are the building blocks of GAGs, i.e., hyaluronic acid, heparin/heparan sulfate, chondroitin sulfate, dermatan sulfate and keratan sulfate [34]. The attachment of GAGs to the protein core results in the synthesis of small proteoglycans (e.g., decorins, fibromodulins, biglycan, testican, bikunin) or larger structures such as aggrecan, brevican, versican and perlecan [36].

One of the most dynamic types of glycosylation is *O*-GlcNAcylation, i.e., the attachment of a single GlcNAc residue to Ser or Thr residues in the polypeptide chain. The attachment of GlcNAC is catalyzed by O-GlcNAc transferase (OGT) and can be reversed by O-GlcNAcase (OGA). Furthermore, Ser and Thr residues are also sites of protein phosphorylation, creating complex cross-talk interactions between these two post-translational modifications of proteins [37].

The structures of all the glycans or antigens described above are shown in Figure 2.

### 2.2. Characteristic Glycan Structures and Their Functional Roles in EV Biology

As a result of the sorting mechanism, EVs from various sources display unique molecular profiles with several glycoepitopes that can be distinguished. The characteristic patterns of EV glycosylation suggest the existence of glycan-based sorting of proteins into EVs. To date, the involvement of complex-type *N*-glycan in EV protein sorting has been the most extensively studied. A study using OVMz cells and kifunensine (an α-mannosidase I inhibitor that prevents the processing of high-mannose-type *N*-glycans to complex and hybrid types) showed reduced levels of *N*-linked glycoproteins (CD63, galectin-3-binding protein (LGALS3BP), a transmembrane protein member of the L1 protein family (L1CAM)) in EVs after treatment, but no change in the expression of no *N*-glycosylated annexin-I [38]. Another α-mannosidase I inhibitor deoxymannojirymycin (DMJ) decreased glycoprotein EWI-2 levels in EVs, and a similar effect was observed after genetically engineered *N*-glycosylation site abrogation. In the same study, enrichment of exosomes with high-mannose glycans, poly-*N*-acetyllactosamine structures and α2,6-linked sialic acids was also observed [39]. Moreover, our previous study using lectin blotting revealed an abundance of β1,6-branched tri- and/or tetra-antennary complex-type *N*-glycans in EVs derived from both normal HCV-29 and cancerous T-24 bladder cells. These glycans were preferentially recruited to EVs despite DMJ treatment. We also demonstrated that EVs derived from cutaneous and uveal melanoma are enriched in glycans with bisecting GlcNAc [40,41]. Regarding sialylation of EV proteins, our previous study demonstrated an enrichment of uveal melanoma-derived EVs in α2,6-linked sialic acid [41]. It has also been shown that glycans bearing α2,6- as well as α2,3-linked sialic acids were preferentially recruited to EVs by cancerous bladder T-24 cells, but not by non-transformed HCV-29 cells. Furthermore, overexpression of α-(1,6)-fucosyltransferase (FUT8) in prostate cancer cells reduces the number and alters the proteome of released EVs [42], including an increased number of proteins associated with cell motility and cancer metastasis. Fucosylated glycans were also preferentially sorted into microvesicles released by cutaneous melanoma cells [40]. The results of the aforementioned studies suggest that the role of glycan structures in EV protein sorting is cell-type-specific rather than uniform. The sorting of particular proteins into EVs may be regulated by different glycan structures, and the glycan-based sorting mechanism may not apply to the entire protein EV cargo but instead to certain groups of proteins.

Besides protein sorting, there is evidence that glycosylation may also affect EV biodistribution, uptake, and effects exerted by EVs on recipient cells. The enzymatic desialylation of EVs with neuraminidase led to their increased accumulation in mice lungs and lymph nodes compared to control EVs [43]. Furthermore, EVs isolated from hepatic cell lines after desialylation were more efficiently internalized by recipient cells, including ovarian cancer cells [44]. Moreover, macrophages showed preferential uptake of α-2,3-sialylated EVs but not neuraminidase-treated EVs [45]. On the other hand, the desialylation of glioblastoma-derived EVs led to their increased uptake by dendritic cells and the activation of CD8+ and CD4+ T cell responses [46]. Analogous observations were made for melanoma-derived EVs overexpressing high-mannose-type glycans (at the expense of sialylated glycans) [47]. Aberrant glycosylation may also contribute to the functional effect exerted by EVs on recipient cells. For example, EVs derived from HCV-29 and T-24 cells have been shown to increase the viability and motility of recipient HCV-29 and T-24, as well as endothelial HUVEC cells and Hs27 fibroblasts [48]. Stronger pro-proliferative and pro-migratory activity of tumor-derived T-24 EVs was observed compared with EVs from normal HCV-29 cells. Furthermore, when EV-releasing cells were pretreated with DMJ, the aforementioned effect was weakened, suggesting that EV glycans are involved in the modulation of recipient cell function. Since β1,6-branched complex-type *N*-glycans and *N*-glycans with α-2,6-linked sialic acids were most abundant in EVs, they were most likely responsible for the observed functional effects. Finally, the glycosylation status of particular EV proteins may determine the effect that EVs have on recipient cells. EVs from breast cancer cells induced recipient cell invasiveness via the transfer of the extracellular matrix metalloproteinase inducer (EMMPRIN) [49]. The observed effect depended on the status of EMMPRIN *N*-glycosylation sites (Asn160 and Asn268). Similarly, EVs bearing the CD82 protein reduced the adhesiveness of recipient ovarian cancer cells depending on CD82 *N*-glycosylation at Asn157 [50].

## 3. Isolation of Urinary EVs

**Figure 3:**
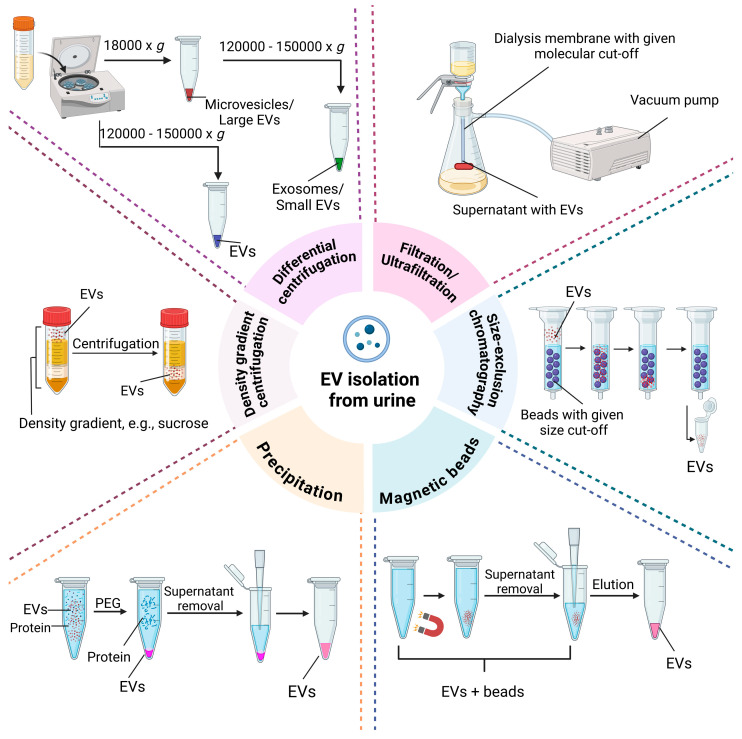
Overview of main methods used for EV isolation.

The use of quality-standardized techniques for the isolation of high-purity EVs from urine is a key factor in the credibility of a study. High levels of protein impurities present in urine samples can interfere with the assessment of EV-derived biomarkers (false positive/negative signals). Furthermore, obtaining an efficient concentration of EVs from small volumes of urine and introducing EV-based diagnostic methods into wide clinical use are other important aspects [51]. The most frequently mentioned methods for the isolation of EVs from urine include filtration, precipitation, magnetic beads, size-exclusion chromatography (SEC), differential centrifugation (DC) and differential centrifugation combined with filtration (DCF) [52] (Figure 3). Nevertheless, new methods for the isolation of EVs are constantly being developed, some of which are based on their glycosylation status.

### 3.1. Commonly Used Methods for EV Isolation from Urine

DC is the most commonly used method for the isolation of EVs from various bodily fluids and conditioned cell culture media. DC does not require expensive chemicals and is a fairly simple procedure. However, it is a multistep process that often results in significant losses of EVs and requires access to an ultracentrifuge to obtain exosomes. The initial volumes of fluids and media, which can often exceed 250 mL, also pose a challenge. Nevertheless, DC is often used to isolate EVs from urine [53,54]. Isolation begins with low-speed centrifugation to eliminate cells and cellular debris, followed by subsequent centrifugation at higher centrifugal forces (up to 150,000–200,000× *g*) to pellet EVs from the supernatant [51,55]. However, it has been shown that even after ultracentrifugation (UC) at 200,000× *g*, approximately 40% of urinary EVs are still not pelleted [56]. Therefore, it is important to analyze whether there are any molecular differences between samples of pelleted urinary EVs and non-pelleted remnants [57]. Moreover, it has been reported that EV pellets obtained by DC may be contaminated with DNA, which should be digested with DNase [58].

Filtration/ultrafiltration techniques are often mentioned as alternatives to DC. However, centrifugation is often necessary in the initial steps to remove cells and cellular debris. Membrane filters with nanosized pores used for ultrafiltration have a molecular mass cut-off of approximately 100 kDa [51]. Prior to the processing of urine samples, membrane concentrators (e.g., Vivaspins) are washed to eliminate glycerol and preservatives [59]. Then, urine is added to the membrane concentrators, and after centrifugation, EVs are recovered from the membrane. However, some recovery buffers used to remove EVs contain dithiothreitol (DTT), which is not compatible with, for example, mass spectrometry-based analytic techniques [59]. Another disadvantage of ultrafiltration is the retention and concentration of soluble urinary proteins with EVs [60]. These proteins also obstruct membrane nanopores and reduce ultrafiltration efficiency [55]. However, this obstacle can be overcome by using membranes with low protein-binding capacity (e.g., hydrophilic polyvinylidene difluoride membranes) [61].

The aforementioned nanopore membranes, which are used to create physical barriers during size-based EV separation, have gained popularity due to their multidimensional applications [62]. Exodisc is a tool for rapid EV isolation and quantification using a lab-on-a-disc integrated with two nanofilters. It enriches samples in EVs with diameters ranging from 20 to 600 nm. Using this technique, high-efficiency isolation of EVs expressing CD9 and CD81 from the urine of patients with bladder cancer has been demonstrated [63]. ExoTIC (exosome total isolation chip) is another EV isolation tool based on membrane filtration. ExoTIC isolates EVs by washing out free nucleic acids and proteins through the nanomembrane filter. The EV yield obtained by this technique is 4 to even 1000 times higher compared to that of DC. It can also sort heterogeneous populations of EVs derived from conditioned culture media, plasma, urine and lavage based on their size. In addition, ExoTIC has been used for the efficient isolation of EVs from small sample volumes (10–100 μL) [64]. Various studies have reported a similar integrated double-filtration microfluidic device that isolates EVs (30–200 nm in diameter) from urine and quantifies them using a microchip ELISA. Receiver operating characteristic (ROC) analysis demonstrated that this integrated EV double-filtration device had a sensitivity of 81.3% with a specificity of 90% [65]. Therefore, multiple filtration-based techniques, especially highly automatized ones, could be used in the near future to fully exploit the potential (mainly diagnostic) of EVs isolated from urine.

Filtration is also part of another EV isolation method based on low-vacuum filtration (LVF) combined with ultracentrifugation. LVF is performed on a dialysis membrane with a molecular weight cut-off of 1000 kDa. After preliminary centrifugations are performed to remove cells and cellular debris, the sample is filtered under a low vacuum (approx. 0.3 bar) and then washed with water [66]. After filtration, the concentrated sample (approx. 2 mL) is centrifuged at 18,000× *g* to obtain an MV-enriched pellet or at 150,000× *g* to obtain an exosome-enriched pellet [67,68]. One of the advantages of this technique is the possibility of concentrating samples before further processing without time-consuming multistep centrifugation.

Furthermore, it has been shown that EVs can be precipitated with the use of polyethylene glycol (PEG) [69,70]. PEG has been used effectively for over seventy years to concentrate and purify viruses [71], which have similar biophysical properties to EVs. Although commercial precipitation reagents (e.g., ExoQuickTM from Systems Biosciences (Palo Alto, CA, USA) and Total Exosome IsolationTM from Life Technologies (Carlsbad, CA, USA)) are easy to use, their prices are prohibitively high, especially when screening multiple samples [69]. However, the method presented by Rider et al. [69] uses PEG solutions added to harvested conditioned cell culture media. After overnight refrigeration, the procedure is followed by medium-speed centrifugation [69]. The same method can be used to isolate EVs from urine. However, one of the key drawbacks of this method is the presence of albumin and other protein contaminants in EVs derived from urine samples [72].

Density gradient centrifugation (DGC), with sucrose and iodixanol used as density media, is also used to isolate EVs from various types of body fluids and conditioned cell culture media. With this technique, isolation can depend on particle size and mass density (top-down gradient) or only on mass density (bottom-up gradient) [73]. In addition, it has been shown that the centrifugation of EVs further down in density gradients improved isolation efficiency compared to that of those floating up in the gradients after the pelleting of EVs with high force [74]. EVs can be analyzed directly or after removal of the density media. Sucrose is removed by dialysis, and iodixanol is removed by dilution followed by EV pelleting at 100,000× *g* [75]. After DGC, EV recovery ranges from 10% to 50%, depending on the removal of the density medium from the sample. Urinary EV isolation with DGC results in low levels of contaminants but is time-consuming and has low throughput [73].

Numerous studies have shown that SEC is one of the most efficient methods for isolating high-quality and high-purity EV samples from urine [52,76,77]. SEC typically requires three-step sample preparation before chromatography. The urine must be centrifugated at low speed to remove cells, followed by centrifugation at medium speed to remove larger vesicles. Final centrifugation is performed at a speed of approx. 17,000× *g* [77]. After the sample is concentrated (often with the use of centrifugal filters), SEC is performed. In a study on optimizing the isolation of EVs from small volumes of urine (0.5 mL and 1 mL), three techniques were compared, i.e., DC, SEC and a proprietary magnetic bead-based commercial method. EVs were characterized using cryogenic electron microscopy, nanoparticle tracking analysis and immunoblotting. To analyze the protein content of EVs, mass spectrometry was performed. Considering the EV yield, the lack of contamination and the quality of proteomic data, SEC was shown to be the optimal method for isolating EVs from small volumes of urine [76]. One of the limiting factors is the fact that SEC (like DGC) is only suitable for input sample volumes no larger than a few milliliters [73].

Finally, total extracellular vesicles recovery and purification (EVTRAP) is a technique based on functionalized magnetic beads, allowing fast and reproducible capture and isolation of EVs from urine samples. The mechanism of EVTRAP is based on the combination of hydrophilic and lipophilic groups with a unique affinity towards the EV lipid bilayer [78]. EVTRAP enabled the capture and improved the extraction of proteins for mass spectrometry. Studies have shown that more than 16,000 unique peptides can be detected using 200 μL of urine [78]. A comparison of EVTRAP with DC showed that during centrifugation, a high amount of urine protein is pelleted. During EVTRAP, the vast majority of the contaminating proteins remain unbound in the supernatant fraction after completed EV isolation [78].

Regardless of the applied isolation method, each EV sample obtained requires thorough standardization and characterization. Before isolation, different qualities of EV sources should be defined, such as the number of releasing cells (if a conditioned medium is used) or, in the case of urine and other biofluids, its input volume and collection/handling methods. Post-isolation samples should be tested for the presence of non-vesicular components (remaining cells, organelles, cell debris) by, for instance, transmission electron microscopy imaging. Simultaneously, the abundance of EVs in the sample should be analyzed in terms of particle numbers (e.g., nanoparticle tracking analysis, flow cytometry) as well as protein/lipid content (e.g., protein concentration assays, Fourier-transform infrared spectroscopy (FTIR)). Finally, the expression of chosen protein markers can be analyzed by immunoblotting or flow cytometry. However, no universal marker specific to EVs regardless of their source is known. Also, biogenesis-related biomarkers (annexin 1, Arf 6 for ectosomes; CD9, CD63, CD81, Lamp1 for exosomes) should be used with caution, since exosomes/ectosomes do not completely overlap in molecular composition [19].

### 3.2. EV Isolation from Urine for Glycomic Studies

The method used for EV isolation can affect not only the vesicle yield and the purity of the sample, but also the results of subsequent experiments, including studies on their glycosylation. Lectins are molecules that bind to carbohydrate structures on the cell surface or elsewhere, and they can be used to detect specific glycoprotein structures that serve as markers. Gerlach et al. [79] compared the lectin-binding pattern in the microarray for EVs isolated from urine by UC or by centrifugal filtration. For most lectins, UC-isolated urinary EVs bound more strongly to lectins than EVs isolated by the latter methods [79].

Lectins were also used to develop the first glycosylation-based method for EV isolation from urine [80]. The study used lectin- and antibody-conjugated beads to capture EVs. The panel of six lectins included jacalin *Artocarpus integrifolia* (AIA) agglutinin, peanut *Arachis hypogaea* agglutinin (PNA), osage orange *Maclura pomifera* (MPA) agglutinin, *Maackia amurensis* (MAA) agglutinin, potato *Solanum tuberosum* (STA) agglutinin and spindle tree *Euonymus europaeus* (EEA) agglutinin, while antibodies against urinary EV markers included aquaporin 2 (AQP2), CD63 and CD24. Populations of EVs isolated from the same urine volumes by lectin binding or antibody binding differed in size and particle count. Interestingly, a higher number of particles did not correlate with a higher protein yield in the obtained samples. As expected, EVs isolated from urine by lectin-conjugated beads exhibited differences in surface glycosylation (revealed by plate microarray) related to lectin specificity [80]. This suggests that lectin-based EV isolation methods can be used to enrich the EV samples in particular glycoprotein groups.

Moreover, if the glycosylation of urinary EVs is analyzed by the most advanced methods, particularly mass spectrometry, such studies are challenged by the low abundance of glycopeptides in the samples and their poor ionization. To address this issue, a novel magnetic hydrophilic material created from ultra-thin two-dimensional molybdenum disulfide with Fe_3_O_4_ nanoparticles, gold nanowire and glutathione (MoS_2_-Fe_3_O_4_-Au/NWs-GSH) was invented [81]. Despite its nanoscale property, it provides a large surface area for efficient hydrophilic interaction-based retention and enrichment of isolated EV glycopeptides prior to MS analyses. The use of MoS_2_-Fe_3_O_4_-Au/NWs-GSH for the analysis of human urinary and serum EVs allowed for the identification of 1250 and 489 *N*-glycopeptides, respectively [81]. Another nanomaterial for the simultaneous capture and enrichment of urinary EVs in glycosylated and phosphorylated peptides was designed by Xiong et al. [82]. Glutathione-functionalized thioether covalent organic frameworks (Fe_3_O_4_@Thio-COF@Au@GSH) utilized hydrophilicity and the switchable charge of GSH to capture exosomes by binding glycoproteins and phosphoproteins on their surface. As a result, a total of 419 glycopeptides (and 316 phosphopeptides) were identified through subsequent LC-MS/MS analysis. The large data set obtained in both studies [81,82] indicates that similar nanomaterials for the enrichment of *N*-glycopeptides hold great promise for the further development of studies related to the urinary EV glycoproteome.

## 4. General Characteristics of Urinary EV Glycome

**Figure 4:**
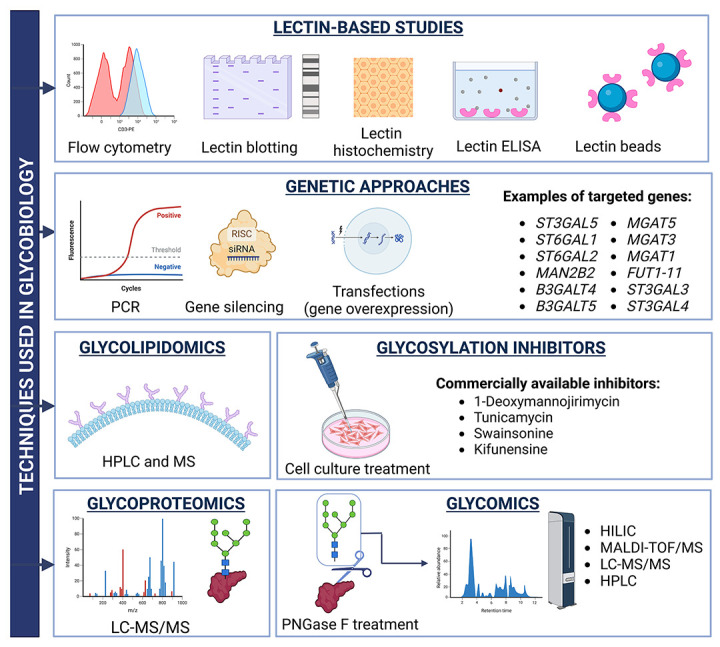
An overview of the main methods used to characterize the EV glycome and glycoproteome.

There are several variously advanced techniques that enable the characterization of urinary EV glycome and glycoproteome both structurally and functionally (Figure 4). The main ones include gas chromatography (GC) and liquid chromatography (LC), which separate glycans based on their physical properties. Both GC and LC can be coupled with mass spectrometry (MS) to identify (and quantify) not only all of the glycopeptides but also glycosylation sites based on the mass-to-charge ratio (*m/z*) of fragmentation ions. To date, the greatest advances in MS-based techniques for glycomic studies have been the development of matrix-assisted laser desorption/ionization (MALDI) and electron spray ionization (ESI). MALDI-MS involves simple sample preparation and is characterized by rapid detection and high sensitivity. However, all of the aforementioned techniques are quite expensive, time-consuming and require sophisticated data analysis. Alternative methods involve the use of glycan-specific antibodies and lectins that recognize sugar residues or entire epitopes. Both antibodies and lectins can be adapted to most of the major biochemical techniques, such as Western blotting, flow cytometry, ELISA or histochemistry. However, those methods show relatively low accuracy, as lectin/antibody binding to the glycans of interest can be inhibited/interfered with by other glycan structures [32]. With regard to functional studies, the use of genetic engineering (knock-outs, gene silencing, transfections) to target, for example, various glycosyltransferases or glycosidases is well documented [83]. Less advanced studies also use glycosylation inhibitors that target different steps of glycan biosynthesis [84]. The function of such artificially altered glycans is then evaluated through various assays/experiments planned for a given study.

Research on urinary EV glycosylation began to develop in the early 2010s. In the first study, Gerlach et al. [79] compared the glycosylation profiles of urinary EVs from healthy volunteers and purified uromodulin (the most abundant soluble glycoprotein in urine) using 43-lectin-panel-based microarray and flow cytometry analyses. The resulting urinary EVs’ glycosylation profiles were similar between samples (including no differences between male and female samples) but differed significantly from the glycosylation profile of purified uromodulin, which bound a limited number of lectins compared to EVs [79]. Similarly, Kosanović et al. [85] used a lectin microarray to obtain urinary EV glycoprofiles. EVs showed an abundance of high-mannose- (reaction with Jack bean *Canavalia ensiformis* (ConA) lectin) or complex-type non-core fucosylated *N*-glycans (lentil *Lens culinaris* (LCA) lectin). A positive reaction for red kidney bean *Phaseolus vulgaris* erythroagglutinin (PHA-E) confirmed the presence of bisected GlcNAc. A weak reaction was observed for red kidney bean *Phaseolus vulgaris* leucoagglutinin (PHA-L), suggesting a low abundance of multi-antennary β1,6-branched complex-type *N*-glycan. Regarding sialylation, α2,6-linked sialic acid (recognized in elderberry bark *Sambucus nigra* agglutinin (SNA)) was superior to α2,3-linked sialic acid (recognized in MAA). Finally, the presence of hexosamine/hexose/lactosamine structures was confirmed in the reaction with hairy vetch *Vicia villosa* agglutinin (VVA), horse gram *Dolichos biflorus* agglutinin (DBA), PNA, castor bean *Ricinus communis* agglutinin (RCA) and wheat germ *Triticum vulgaris* agglutinin (WGA). Positive reactions with these lectins may also indirectly suggest the presence of *O*-linked glycans, including Tn antigen, T antigen and blood group A antigen [85].

Shortly after, the results of more detailed structural glycomic and glycoproteomic analyses of urinary EVs from healthy individuals were published [86]. The first study analyzed intact *N*-glycopeptides (including peptide sequences, glycan compositions, structures and glycosylation sites) by collision-induced dissociation MS/MS (CID-MS/MS), and released glycans from the same samples by classical MALDI-MS. CID-MS/MS identified 126 *N*-glycopeptides with 51 *N*-glycosylation sites, which added up to 37 glycoproteins, whereas *N*-glycan compositions were found by MALDI-MS. The identified glycoproteins were mainly of extracellular, cytoplasmic and membrane origin according to Gene Ontology analysis. Most of them possessed one *N*-glycosylation site, except for aminopeptidase N, megalin (three sites) and cubilin (five sites). Regarding the *N*-glycan structures found in urinary EVs, 82% were of the complex type (including 38% with bisecting GlcNAc, 50% sialylated, 65% fucosylated), 14% of the high-mannose-type and 3% of the hybrid-type [86].

A similar MALDI-MS-based study also revealed an abundance of high-mannose- and complex-type glycans (extensively fucosylated and sialylated) in urinary EVs [87]. Interestingly, paucimannosidic glycans were also identified. Although such simple structures are characteristic of plants or invertebrates, their presence has been confirmed in human embryonic stem cells, buccal epithelial cells, colorectal cancer cells, and various immune cells during inflammation. This study was also the first to analyze *O*-glycans in urinary EVs. The structures commonly identified in the samples included Tn (GalNAc), sialylated Tn and extended core 2 Galβ1–3(GlcNAcβ1–6)GalNAc [87]. This suggests that *O*-glycans can also be extracted from urinary EVs in amounts that allow for further structural analyses.

Recently, another glycoproteomic study was performed on urinary EVs from healthy volunteers [88]. A total of 3144 unique glycosylation events, 378 glycoproteins and 604 glycosylation sites were identified, establishing the EV glycoproteome microheterogeneity as 5.9 glycans per site. It was also observed that if a urinary-EV-derived glycoprotein bears highly abundant glycan, this correlates with a smaller number of different glycans on this glycoprotein. In contrast, less abundant glycans are more likely to be found on heterogeneously glycosylated proteins. Nevertheless, the most abundant glycans HexNAc_2_Hex_5_ and HexNAc_2_Hex_6_ (Hex = hexose; HexNAc = *N*-acetylohexosamine) were found on proteins bearing an average of 21.2 and 22.7 glycans, respectively [88].

Finally, protein post-translational modifications (PTMs) observed in urinary EVs include not only glycosylation but also phosphorylation. Both modifications occur concurrently in cells and can affect protein sorting into EVs and EV biological function. Zheng et al. [89] developed hydrophilic carbonyl-functionalized magnetic zirconium-organic (CFMZOF) probes to capture peptides from isolated urinary EVs bearing these two PTMs. They used three fractions of urinary EVs isolated by SEC, i.e., large (L-Exo, mean diameter ~102 nm), medium (M-Exo, mean diameter ~60 nm) and small (S-Exo, mean diameter ~36 nm) exosomes. Subsequent LC-MS/MS analyses led to the identification of 144 glycoproteins and 44 phosphoproteins in L-Exo, 156 glycoproteins and 46 phosphoproteins in M-Exo, and 134 glycoproteins and 10 phosphoproteins in S-Exo. The percentage of proteins with simultaneous glycosylation and phosphorylation was 11%, 9% and 3% for L-Exo, M-Exo and S-Exo, respectively. Finally, principal component analysis revealed that particular exosome fractions were highly heterogenous in terms of the abundance of particular glyco- and phosphoproteins. L-Exo were enriched in PTM-bearing proteins related to immune response, M-Exo in those related to biological metabolism and S-Exo in those related to molecule transport processes [89]. This suggests that PTMs of urinary EV proteins contribute significantly to their size-dependent heterogeneity.

## 5. Urinary EV Glycome in Various Disease States

Research on EV glycosylation has clinical significance and has garnered increasing attention in recent years. Once delivered to recipient cells, EVs modulate multiple biological processes, including those associated with the development and progression of various diseases. Moreover, the glycosylated constituents of EV cargo undergo dynamic changes reflecting the current states and molecular content of the releasing cells. The EV glycome is therefore considered as an excellent source of glycobiomarkers [31,32]. The following sections summarize the available data on the involvement of the urinary EV glycoproteome in several cancers, as well as in metabolic, cardiovascular, neurological or kidney diseases.

### 5.1. Glycosylation of Urinary EVs in Cancer

#### 5.1.1. Prostate Cancer

Urine from prostate cancer patients is more abundant with EVs than urine from patients with benign prostate hyperplasia. Furthermore, EVs isolated from prostate cancer patients’ urine have increased diameter and molecular density. It has also been shown that rectal massage increases the number of EVs present in the urine of prostate cancer patients and the likelihood that EVs present in urine originate from the prostate [90].

An analysis of urine *N*-glycosylation showed that overall core fucosylation and bi-antennary glycosylation were higher in patients with benign prostate hyperplasia than in patients with prostate cancer [91]. Core fucosylation was used to create a urinary glycoprofile marker (UGM), which is obtained by dividing the ratio of nonfucosylated bi-antennary, tri-antennary and tetra-antennary structures by the sum of the total number of tri-antennary glycan structures and the prostate volume [92]. Vermassen et al. [91] showed that the UGM helped to distinguish prostate cancer from benign prostate hyperplasia [91]. Whether the use of UGM is feasible for EVs has not yet been determined.

In addition, prostate-specific antigen (PSA) is a glycoprotein with a single *N*-glycosylation site (Asn-69) and is one of the main diagnostic tools for prostate cancer [93]. MALDI-TOF profiling of PSA glycosylation constituents identified 40 *N*-linked glycan structures of partially purified PSA from seminal plasma of control, benign disease and prostate cancer donors [94]. It was also noted that urinary total PSA (tPSA) concentration decreases after N-butanol extraction of vesicle-associated PSA. The PSA extraction rate was lower in patients with benign prostate hyperplasia. It was demonstrated that the urinary tPSA level after vesicle-associated extraction was correlated with the amounts of biantennary structures and bi-antennary core-fucosylation [91].

Another study on the glycoprofiles of prostate cancer patients showed an increased expression of α(1,6)fucosyltranferase (FUT8), which catalyzes the addition of core fucose to *N*-glycans. Overexpression of FUT8 was associated with increased tumor invasiveness, aggressiveness and castration resistance [95,96,97]. Overexpression of FUT8 in prostate cancer cell lines was also shown to reduce EV release, but it did not affect vesicle size [42]. In the same study, proteomic analysis of EVs derived from the LAPC4 cell line with and without induced FUT8 overexpression showed that EVs derived from control LAPC4 cells were more enriched in endosome-derived proteins (e.g., clathrin-mediated endocytosis components and endosomal sorting complex required for transport (ESCRT) complex components). These results may indicate that overexpression of FUT8 in prostate cancer cells disturbs processes related to endocytosis or even exosome formation, as the ESCRT complex is essential for their biogenesis. A subsequent *N*-glycosylation analysis identified 124 intact glycopeptides that were more abundant in control LAPC4-derived EVs, whereas 103 intact glycopeptides were overexpressed in EVs from FUT8-overepressing cells. Furthermore, opposite compositions of high-mannose and fucosylated glycan were observed between the two EV populations [42]. Finally, overexpression of FUT8 resulted in much more variable glycoside occupancy and glycan composition at the same glycosylation sites on EV glycoproteins [42]. Therefore, based on the promising results of in vitro studies, the FUT8 content in urinary EVs should become one of the areas of interest for future studies.

Moreover, expressed prostatic secretions (EPSs), alternatively known as post-digital rectal exam urines, are fluids originating from the prostate gland. EPSs are widely used in diagnostic and prognostic evaluations for prostate cancer [98,99]. Previously, EPSs have been shown to be enriched in more than 1400 individual proteins [100,101,102]. Furthermore, proteomic studies of EPS fluids obtained during prostatectomies have shown that six of the seven most abundant proteins are *N*-glycosylated, i.e., immunoglobulins, lactotransferrin, prostatic acid phosphatase (PAP), zinc alpha-2 HS glycoprotein, aminopeptidase N and PSA [100].

In addition, a matrix-assisted laser desorption/ionization Fourier-transform ion cyclotron resonance mass spectrometry (FT-ICR MALDI) study revealed distinct differences in the glycosylation profiles of exosomes isolated from prostate cancer samples compared to non-cancer samples. EPS-derived exosomes from prostate cancer patients were abundant with a bisecting GlcNAc-hybrid structure of Gal_1_N_2_M_5_N_2_ (Gal = galactose; M = mannose; N = *N*-acetylohexosamine) and had a higher proportion of tetra-antennary glycans [102]. Similarly, a MALDI technique was used to analyze the *N*-glycosylation profiles of samples of EPS urine (EPSu), direct EPS fluids (EPSd) and EPS-derived EVs (EPSev). The abundance of *N*-glycan classes varied across the sample groups. The most abundant *N*-glycans were typically bi-antennary with two galactoses in EPSd, and EPSev showed a similar *N*-glycan composition, with most of the *N*-glycans being bi-antennary and/or fucosylated. However, EPSev had higher amounts of high-mannose *N*-glycans. EPSu *N*-glycans had about 20% less sialylation than *N*-glycans from EPSd and EPSev, but an increased amount of tetra-antennary and sulfated *N*-glycans. Finally, the EPSd and EPSev samples had higher levels of *N*-glycans with sialic acids and no fucose compared to EPSu [103]. Therefore, EPSs, rich in glycoproteins and EVs, have significant diagnostic potential.

Furthermore, Islam et al. [104] described a nanoparticle-based approach for the detection of EVs derived from urine and a cell culture supernatant of LNCaP cells. The nanoparticle-based time-resolved fluorescence immunoassay (NP-TRFIA) uses biotinylated antibodies against tetraspanin family proteins (e.g., CD9, CD63 and CD81) or lectins against specific glycoepitopes to capture EVs without the need for isolation [104,105]. The EV detection threshold using nanoparticles with C-type DC-SIGN lectin or mannose-binding lectin (MBL) was found to be as low as 1–2 ng/mL, almost 3-fold lower than that of nanoparticles conjugated with CD9 or CD81 antibodies. Therefore, this technique can be used to identify disease-specific glycomarkers on the surface of urinary EVs [104].

Finally, glycosphingolipids may also be a glycoconjugate family of interest regarding urinary EVs in prostate cancer. Exosomes derived from PC3 cells showed an enrichment of glycosphingolipids (alongside sphingomyelin, cholesterol and phosphatidylserine) more than 15 times higher than PC3 cells. These enriched glycosphingolipids included xexosylceramide, lactosylceramide, globotriaosylceramide and gangliosides (GD1, GM1, GM2 and GM3) [106]. The mentioned glycosphingolipids are present in the outer membrane of exosomes; therefore, they are easily accessible to antibodies and can be used as diagnostic and prognostic markers in prostate cancer. Moreover, exosomes derived from PC-3 cells have been shown to have an 8.4-fold enrichment of lipids per mg of protein [106]. Furthermore, the lipid-to-protein ratio of 8.4:1 in PC-3-derived exosomes suggests that glycolipids are a major reservoir of EV glycans rather than proteins [106,107,108].

#### 5.1.2. Bladder Cancer

EVs released by bladder cancer cells can also be found in urine. In our previous studies, we demonstrated that microvesicles derived from bladder cancer T-24 cells and normal bladder epithelial HCV-29 cells exhibit distinct total and surface glycosylation profiles with an abundance of β-1,6-branched and sialylated glycans. Microvesicles derived from T-24 cells showed stronger pro-proliferative and pro-migratory effects compared with those from HCV-29 cells. These effects were reduced when microvesicles were isolated from cells treated with an α-mannoside II inhibitor, i.e., 1-deoxymannojirymycin (DMJ), indicating that glycans carried by microvesicles play a role in modulating recipient cell function. In addition, microvesicles from both HCV-29 and T-24 cells increased the viability and motility of endothelial HUVEC cells and Hs27 fibroblasts, supporting the hypothesis that microvesicles can affect the function of different cells in the tumor microenvironment [48]. Although EVs, as a constituent of urine, should be considered as waste products before they end up removed, they most likely circulate throughout the body, exerting their biological effects. Therefore, it would be worthwhile to conduct functional tests with respect to their glycosylation, for example, using specific glycosidases before their incubation with recipient cells.

Furthermore, integrin α3 (ITGA3) is an adhesion molecule which is overexpressed in various types of cancer [109,110,111,112,113,114,115]. ITGA3 is also overexpressed in bladder cancer and correlated with poor prognosis. Suppression of ITGA3 by miR-199 family miRNAs has been shown to lead to tumor suppression [116]. Recently, a non-invasive assay has been described to identify fucosylated-glycoisoform of ITGA3 from unprocessed urine from bladder cancer patients [117]. ITGA3 was detected using *Ulex europaeus* agglutinin-I (UEA-I)-coated nanoparticles. This assay was successfully used to distinguish bladder cancer patients from controls [117]. In later study, UEA-I has been shown to strongly bind to fucosylated ITGA3 on EVs [118]. Finally, in clinical settings, the assay significantly discriminated individuals with bladder cancer from those with prostate cancer, benign prostatic hyperplasia and healthy individuals [119]. This suggests that glycans or glycans present on a particular protein in urinary EVs can be used to design fast, non-invasive and simple diagnostic tests.

#### 5.1.3. Renal Cancer

Renal cancer cells may also be a source of EVs found in urine, and their glycosylation may also contribute to their biological function or diagnostic potential. For example, azurocidin 1 (AZU1) is a chemoattractant for monocytes and macrophages secreted by neutrophils, which facilitate the permeabilization of vascular endothelium. AZU1 has been shown to be abundant in renal cell carcinoma-derived EVs, particularly in those derived from Caki-1 and Caki-2 cell lines. Treatment with tunicamycin, an inhibitor that completely blocks *N*-glycosylation, reduced the molecular weight of AZU1 in cells and the level of AZU1 in EVs. Furthermore, inhibition of *N*-linked glycosylation by introducing mutations at three *N*-glycosylation sites (Asn126→Gln, Asn140→Gln and Asn171→Gln) inhibited AZU1 loading into EVs in ACHN cells. Moreover, EVs released from AZU1 mutant cells could not effectively alter the permeability of the vascular endothelial cell layer, compared with EVs derived from control cells [120]. The presence of glycosylated AZU1 in urinary EVs from renal cancer patients has not been confirmed so far. This should be the subject of future studies to expand knowledge on the role of AZU1 glycosylation, especially in intravasation and extravasation during metastasis.

In addition, circulating RNAs (circRNAs) have been shown to be involved in the development of human diseases, including malignant tumors [121]. CircRNAs can act as microRNA sponges, bind to various proteins, and most importantly, interfere with translation [122]. In renal carcinoma, circRNAs promote cancer progression by interacting with microRNA and mRNA networks [123]. CircSPIRE1 is metastasis-suppressing circRNA, whose expression is reduced in patients with metastatic renal carcinoma and predicts better patient survival. CircSPIRE1 is packaged in exosomes and transferred by them to endothelial azurocidin (AZU1) cells, suppressing vascular permeability and angiogenesis [124]. Furthermore, circSPIRE1 serves as a scaffold between ELAV-like RNA-binding protein 1 (ELAVL1) and *N*-acetylgalactosaminyltransferase 3 (GALNT3). In renal cancer, GALNT3 promotes the glycosylation and cytomembrane localization of E-cadherin (promoting a benign, epithelial phenotype) [124]. This suggests that not only glycoproteins themselves but also molecules involved in the regulation of glycosylation, such as enzymes or RNAs, should be investigated in urinary EV cargo.

Recently, Wang et al. [125] developed the analytical iMAGE (integrated magnetic analysis of glycans in extracellular vesicles) platform for the glycoprofiling of EVs from various body fluids. The iMAGE platform uses rationally designed polycore magnetic nanoparticles to transduce EV-bound glycans, but not free-floating glycoproteins, into magnetic signals. In the next step, the obtained signals are readily quantified by a built-in magnetoresistance sensor. The advantages of iMAGE include its short time duration (<30 min), its sensitivity (<104 EVs) and the fact that no washing is required [125]. Moreover, the effectiveness of this strategy was tested using spiking renal cancer cell-derived EVs as well as urinary and serum EV samples.

Finally, the combination of immunomodulatory and drug carrier properties allows for the use of EVs as anticancer vaccines [126]. A mammalian co-expression plasmid of glycolipid-anchored-IL-12 (GPI-IL-12) was used to analyze whether exosomes derived from IL-12-anchored renal cancer cells enhance immunogenicity and antitumor response. These exosomes expressed renal cell carcinoma-associated antigen G250 and GPI-IL-12 and promoted T cell proliferation and increased IFN-gamma release. To summarize, treatment with exosomes enriched with IL-12 could effectively induce antigen-specific cytotoxic T lymphocytes, offering a new exosome-based vaccine strategy for renal cell carcinoma [127]. In this context, it would be interesting to investigate whether urinary EV glycoepitopes, which partially reflect the glycan content of releasing cells (including cancer cells), could help in the development of new anticancer EV-based vaccines.

### 5.2. Glycosylation of Urinary EVs in Non-Cancerous Diseases

#### 5.2.1. Kidney Diseases

Despite constant development of less invasive diagnostic methods, the exact etiology of chronic kidney diseases (CKDs) is still determined mainly by biopsies. Alternative methods for diagnosing and monitoring CKD are therefore highly desirable, and urinary EVs are a potential target. Gerlach et al. [79] used a lectin microarray to obtain urinary EV glycoprofiles from patients with autosomal dominant polycystic kidney disease (ADPKD) and healthy controls. The patients’ EVs showed stronger binding to 6 out of 43 lectins, i.e., AIA lectin (recognizing Gal (sialylation independent)), PA-I (Pseudomonas (*Pseudomonas aeruginosa*)) lectin (recognizing Gal and Gal derivatives), NPA (daffodil (*Narcissus pseudonarcissus*)) lectin (recognizing Man-α(1,6)- epitope), RCA-I lectin (recognizing Gal-β-(1,4)-GlcNAc epitope), AAL (orange peel fungus (*Aleuria aurantia*)) lectin (recognizing α-Fuc (1,6)) and GS-I-B4 (Griffonia/Bandeiraea (*Griffonia simplicifolia/Bandeiraea simplicifolia*)) lectin-I (recognizing α-Gal). Moreover, principal component analysis showed that using the combined data of these six lectins provides the most distinct groups for a comparison of ADPKD and healthy controls [79]. The study revealed that EVs in urine can show disease-specific glycan modifications for CKDs, although testing is still required for CDKs of an etiology other than that of ADPKD.

#### 5.2.2. Neurodegenerative Diseases

Altered glycosylation has been observed in various neurodegenerative diseases. For example, abnormal glycosylation patterns have already been observed in the plasma and cerebrospinal fluid of patients with Parkinson’s disease, but little is known about characteristic glycans present in their urine. Recently, Xu et al. [128] used LC-MS to analyze digested glycans and intact glycopeptides in urine, revealing a depletion of total *N*-glycans (mainly bi-antennary galactosylated *N*-glycans) compared to healthy controls, but also an increased abundance of S(6)_1_H_5_N_4_F_1_, S(6)_2_H_5_N_4_ and N_4_H_4_F_1_ (S = sialic acid; H = hexose; N = *N*-acetylohexosamine; F = fucose) structures. The study also analyzed the numbers and positions of glycosites. Alpha-1-microglobulin/bikunin precursor (AMBP), uromodulin and RNase1 were shown to have specific *N*-glycosylation sites that were missing in healthy controls, while the total number of identified glycosites was reduced in patients with disease. The development of similar studies using urinary EVs may help in the search for more potential glycobiomarkers for Parkinson’s disease [128].

#### 5.2.3. Cardiovascular Diseases

Anti-neutrophil cytoplasmic antibodies (ANCA)-associated vasculitis (AAV) is an autoimmune condition that leads to systemic, necrotizing inflammation of blood vessels, manifesting mainly in the kidneys, lungs and ENT (ear, nose, throat) organs. Most patients with AAV show increased levels of ANCA against proteinase-3 or myeloperoxidase, and untreated patients are at risk of mortality, with a rate of nearly 90% [129]. Because of the severity of AAV, biomarkers for early detection are needed. LC-MS proteomic studies of urinary EVs from AAV patients revealed their enrichment in Golgi enzymes, such as mannosyl-oligosaccharide 1,2-alpha-mannosidase IA (MAN1A1), which are involved in *N*-glycan biosynthesis. *N*-glycans may then participate in T cell activation, a process that likely contributes to AAV development [129]. This shows that not only glycans themselves but also the enzymes involved in various steps of glycosylation are transported within EVs, and can affect the glycosylation occurring in recipient cells. As for EVs in urine, identification of such enzymes in their cargo may provide new markers of AAV.

#### 5.2.4. Metabolic Disorders

Almost every metabolic disease manifests with characteristic changes in urine composition. These changes can include glycoproteomic content, and while changes in total urine glycosylation have been studied many times, data on isolated EVs in urine are still very limited. Staubach et al. published two papers demonstrating a characteristic pattern of urinary EV glycosylation [130] and identifying glycoproteins present in the urinary EV cargo [131] from patients with galactosemia. An analysis of glycans released by PNGase F (Peptide:*N*-glycosidase F) conducted using MALDI-MS showed a significant shift in the ratio of abundance of high-mannose- to complex-type glycans from ~0.25 to 1 in urinary EVs from healthy controls and patients with galactosemia. Interestingly, a similar change was not observed for the most abundant glycoproteins in urine, uromodulin [130]. EVs from the urine of patients with galactosemia were also enriched in serum glycoproteins, including albumin, leucine-rich α-2-glycoprotein, fetuin, immunoglobulins, prostaglandin H2 d-isomerase and AMBP—all closely associated with various types of nephropathies or renal damage [131]. This may be a consequence of impaired renal filtration, leading to the enrichment of exosomal membranes with adsorbed serum glycoproteins.

## 6. Conclusions and Future Perspectives

**Figure 5:**
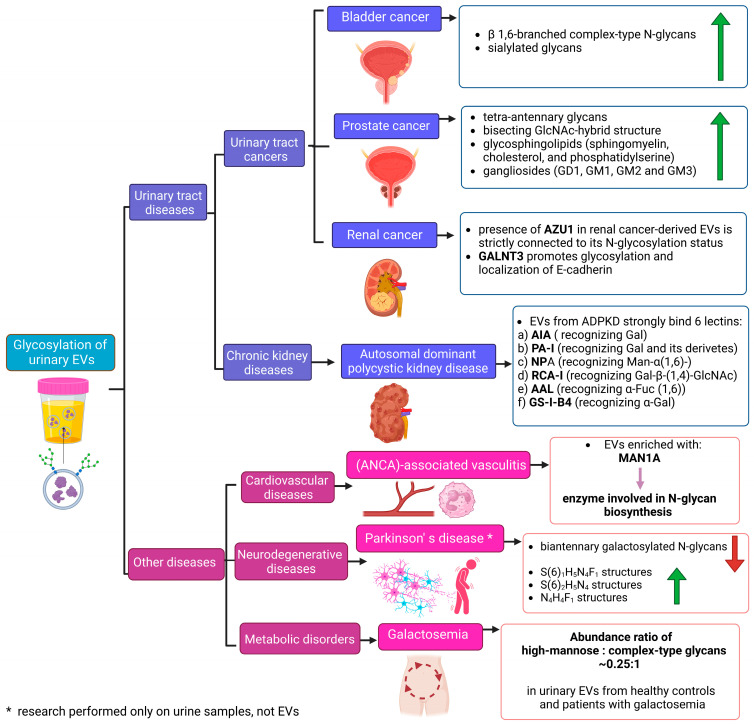
Summary of findings from studies on urinary EV glycosylation, regarding characteristic glycan structures, lectin-binding pattern, abundant glycoproteins, etc. Abbreviations used include the following: AAL—orange peel fungus *Aleuria aurantia* lectin; AIA—*Artocarpus integrifolia* agglutinin; AZU1—azurocidin; F—fucose; GALNT3—N-acetylgalactosaminyltransferase 3; GD1—disialotetrahexosylganglioside 1; GM1/2/3—monosialotetrahexosylganglioside 1/2/3; GS-I-B4—*Griffonia simplicifolia* lectin-I; H—hexose; MAN1A1—mannosyl-oligosaccharide 1,2-alpha-mannosidase IA; N—N-acetylohexosamine; NPA—daffodil *Narcissus pseudonarcissus* lectin; PA-I—*Pseudomonas aeruginosa* lectin; RCA—castor bean *Ricinus communis* agglutinin; S—sialic acid.

Ongoing analyses of urinary EV glycosylation are providing new insights into the role of glycans in EV biology in both physiological and pathological states. Moreover, differentially expressed or unique glycan structures (or entire glycoconjugates) identified in urinary EVs are considered potential biomarkers of urinary tract diseases and several other conditions, including cardiovascular, neurological and metabolic disorders (Figure 5). However, standardized and validated protocols for high-throughput glycomic/glycoproteomic analysis of urinary EVs are still lacking for use in clinical settings. Commonly used LC-MS-based methods have various limitations regarding their accuracy, specificity, detection thresholds and ability to quantify results. As a result, detailed workflows including appropriate EV isolation methods not affecting subsequent glycosylation analysis have not yet been developed. Also, the presence of soluble glycoproteins in urine (such as uromodulin) should be carefully considered in terms of EV sample purity. A standardized glycomic approach to urinary EV analysis could then be applied in studies of not only the diseases described in this article, but also of other conditions whose glycosylation patterns may also be reflected in urinary EV cargo.

## Figures and Tables

**Table 1:** Biomarkers of origin present in urinary EVs.

Organ	Structure/Cell Type	Biomarker of Origin Present in EVs	Reference
Prostate	Epithelial cells	Prostate-specific membrane antigen	[22]
		Prostatic acid phosphatase	[23]
		Prostate transglutaminase	[24]
Bladder	Epithelial cells	Mucin-1, Uroplakin-1, Uroplakin-2	[25]
Kidney	Podocytes of glomerulus	Complement receptor 1	[26]
		Wilm’s tumor 1	[27]
		Canonical transient receptor potential 6, Nephrin, Podocin, Podocalyxin	[28]
	Proximal tubule	Angiotensin-converting enzyme, Aminopeptidase N, Aquaporin 1, Carbonic anhydrase, Megalin	[25]
		Sodium/glucose cotransporter 2	[23]
	Henle’s loop	Aquaporin 1, Na-K-2Cl cotransporter, Uromodulin	[25]
	Distal tubule	Prominin 2, Claudin 1	[29]
		Thiazide-sensitive Na-Cl cotransporter, Aquaporin 2	[25]
	Collecting duct	Mucin 1, Aquaporin	[25]
		Cloudin 1	[29]

## Data Availability

No new data were created during the preparation of this manuscript.

## Abbreviations

AAL	orange peel fungus *Aleuria aurantia* lectin
AAV	ANCA (anti-neutrophil cytoplasmic antibodies)-associated vasculitis
ADPKD	autosomal dominant polycystic kidney disease
AIA	Artocarpus integrifolia agglutinin
AMBP	alpha-1-microglobulin/bikunin precursor
ANCA	anti-neutrophil cytoplasmic antibodies
AZU1	azurocidin 1
circRNA	circulating RNAs
CKD	chronic kidney diseases
CoA	*Canavalia ensiformis* lectin
DBA	horse gram *Dolichos biflorus* agglutinin
DC	differential centrifugation
DGC	density gradient centrifugation
DMJ	deoxymannojirymycin
EEA	spindle tree *Euonymus europaeus* agglutinin
ENT	ear, nose and throat
EPS	expressed prostatic secretions
ESI	electron spray ionization
EVs	extracellular vesicles
EVTRAP	total extracellular vesicles recovery and purification
EXO	exosomes
ExoTIC	exosome total isolation chip
Fuc	fucose
FUT8	α-(1,6)-fucosyltransferase
GAGs	glycosaminoglycans
Gal	galactose
GalNAc	*N*-acetylgalactosamine
GALNT3	*N*-acetylgalactosaminyltransferase 3
GC	gas chromatography
GD	gangliosides
GD1	ganglioside 1
GIP-IL-12	glycolipid-anchored-IL-12
GlcNAc	*N*-acetyloglucosamine
GnT-III	*N*-acetylglucosaminyltransferase III
GnT-V	*N*-acetylglucosaminyltransferase V
GS-I-B4	Griffonia/Bandeiraea (*Griffonia simplicifolia/Bandeiraea simplicifolia*) lectin-I
iMAGE	integrated magnetic analysis of glycans in extracellular vesicles platform
ITGA3	integrin α3
LC	liquid chromatography
LCA	lentil *Lens culinaris* lectin
LC-MS/MS	liquid chromatography coupled with tandem mass spectrometry
L-exo	large exosomes
MAA	*Maackia amurensis* agglutinin
MALDI-TOF	matrix-assisted laser desorption ionization–time-of-flight mass spectrometry
Man	mannose
M-exo	medium exosomes
MPA	osage orange *Maclura pomifera* agglutinin
MS	mass spectrometry
MUCs	mucins
MVBs	multivesicular bodies
NPA	daffodil *Narcissus pseudonarcissus* lectin
PA-I	*Pseudomonas aeruginosa* lectin
PAP	prostatic acid phosphatase
PHA-E	red kidney bean *Phaseolus vulgaris* erythroagglutinin
PHA-L	red kidney bean *Phaseolus vulgaris* leucoagglutinin
PNA	peanut *Arachis hypogaea* agglutinin
PSA	prostate-specific antigen
PTMs	post-translational modifications
RCA	castor bean *Ricinus communis* agglutinin
SEC	size-exclusion chromatography
S-exo	small exosomes
Sial	sialic acid
SNA	*Sambucus nigra* agglutinin
STA	potato *Solanum tuberosum* agglutinin
tPSA	urinary total PSA
UC	ultracentrifugation
UEA-I	*Ulex europaeus* agglutinin-I
UGM	urinary glycoprofile marker
VVA	hairy vetch *Vicia villosa* agglutinin
WGA	wheat germ *Triticum vulgaris* agglutinin
